# Volcanic monitoring of the 2021 La Palma eruption using long-period magnetotelluric data

**DOI:** 10.1038/s41598-023-43326-0

**Published:** 2023-09-23

**Authors:** P. Piña-Varas, J. Ledo, P. Queralt, D. Martínez van Dorth, A. Marcuello, I. Cabrera-Pérez, L. D’Auria, A. Martí

**Affiliations:** 1 Departament de Dinàmica de La Terra I de LˈOceà, Facultat de Ciències de La Terra. Universitat de Barcelona, Barcelona, Spain; 2 Departamento de Física de La Tierra y Astrofísica, Facultad de Física, Universidad Complutense de Madrid, Madrid, Spain; 3 Instituto Volcanológico de Canarias (INVOLCAN), 38600 Granadilla de Abona, Tenerife, Canary Islands Spain

**Keywords:** Geodynamics, Geophysics, Volcanology

## Abstract

Between September and December 2021, the first subaerial volcanic eruption in the Canary Islands in 50 years took place on the island of La Palma. Since November 2021, we have been conducting a long-period magnetotelluric (MT) monitoring experiment at a site located 2.4 km east of the volcanic cone. Having continuously recorded data since then, the obtained dataset shows significant changes in resistivity over the fourteen months following the eruption: more than ± 20% in apparent resistivity and ± 2 degrees in phase. These temporal variations in electrical resistivity, recorded continuously using long-period MT during both the syn- and post-eruptive stages, have not been reported to date, making this dataset unique. Four estimated impedances have been selected as representatives of the major temporal changes observed and inverted to generate new 3-D resistivity models. The results provide novel key information on the spatiotemporal evolution of the subsoil's electrical resistivity, enabling the characterization of a set of structures acting as preferred magmatic fluid pathways. Therefore, our study highlights the strong potential of MT as a volcanic monitoring tool and provides new insights about the evolution of the fluid pathways during the post-eruptive stage. These findings enhance our understanding of the magmatic system and may contribute to volcanic hazard mitigation in the future.

## Introduction

Geophysical methods are essential tools for volcanic monitoring, providing valuable insights into the plumbing system and processes of volcanoes. The control and monitoring of volcanic eruptions is usually carried out by means of seismic and ground deformation methods. Electromagnetic (EM) methods, being sensitive to changes in electrical resistivity, are not commonly used despite their potential as volcanic monitoring tools, as they are sensitive to temperature and the presence of fluids.

Among the EM methods, the magnetotelluric (MT) method is particularly valuable as it provides information on the electrical resistivity of the subsurface across a wide range of depths. Thus, during a volcanic eruption, MT could provide spatial and temporal information about the presence and variations in fluid characteristics as magma rises.

MT has proven successful in characterizing magmatic systems in volcanic environments (e.g.^1–10^,), but only a few studies have focused on detecting changes in the electrical resistivity distribution over time (e.g.^11,12^,).

On September 19, 2021, the first subaerial volcanic eruption in the Canary Islands in the last 50 years began, resulting in a new edifice on the western flank of Cumbre Vieja (La Palma Island). This volcanic eruption lasted for a total of 85 days, offering a unique opportunity to assess changes in electrical resistivity during and after an active volcanic eruption (syn- and post-eruptive stages).

**Figure 1:**
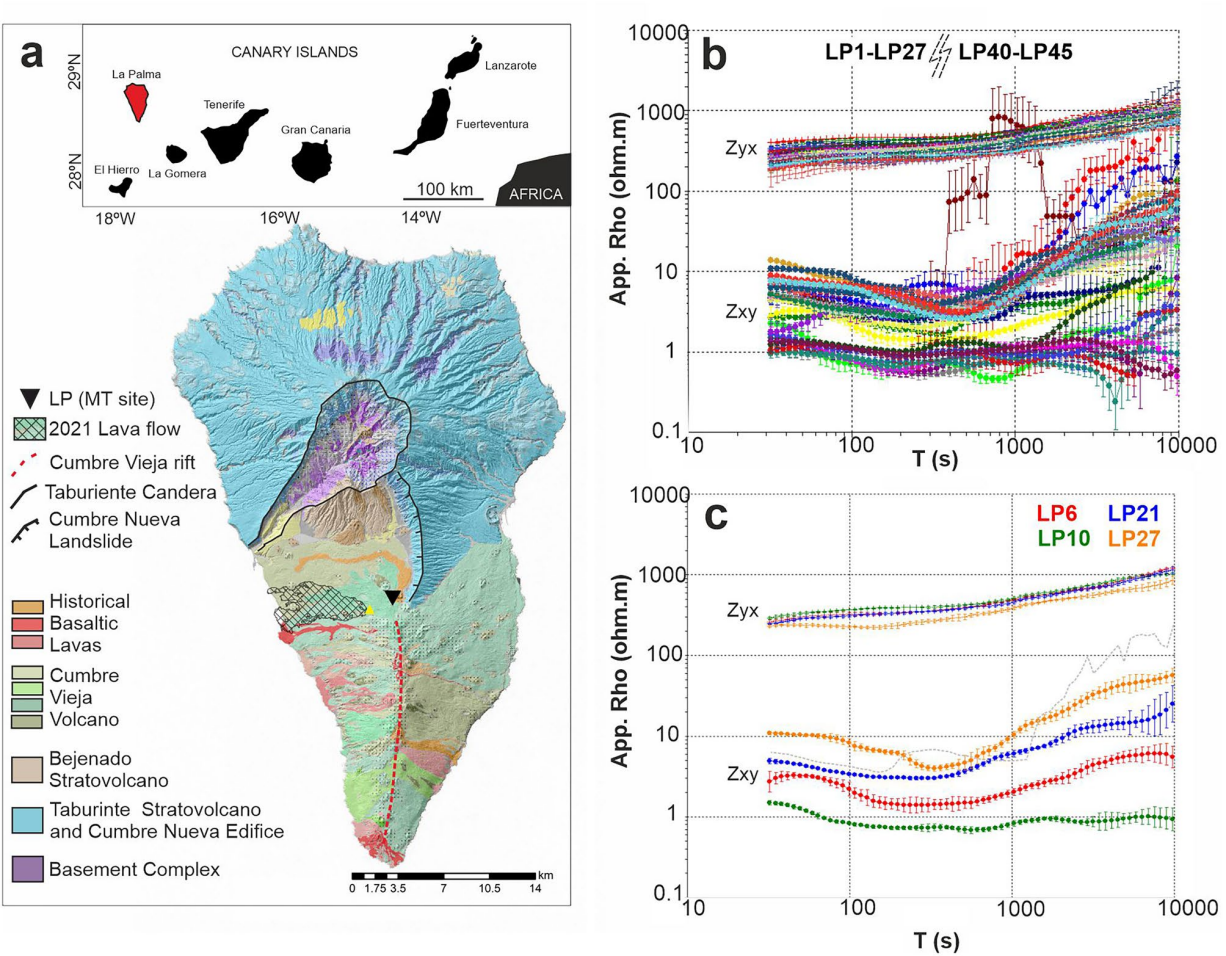
(**a**) Simplified geological map of La Palma Island showing the major geological features. Black triangle: long-period MT site; Yellow triangle: 2021 Cumbre Vieja volcano. (**b**) Off-diagonal apparent resistivity curves for the temporal MT impedances obtained by processing the original time series every ten days. (**c**) Four selected apparent resistivity curves representative of the major temporal resistivity changes. Grey dashed line: LP1 curve. The geological map and detailed legend can be found at https://idecan2.grafcan.es.

Since the start of the volcanic eruption, a comprehensive MT monitoring experiment has been conducted on the island. A long-period MT site (period range: 1 to 100,000 s) was deployed 2.4 km away from the volcanic cone (Fig. 1A). The equipment has been continuously recording data starting from November 5, 2021, when the eruption had already begun. This dataset represents one of the few long-term (over fourteen months) continuous MT datasets recorded on an active volcano, and is notable for not having been recorded using broadband MT, but rather long-period MT. The initial results obtained from this dataset reveal significant changes in electrical resistivity over time. Such temporal variations in electrical resistivity during the syn- and post-eruptive stages of an active volcanic eruption have not been previously reported, making this dataset unique. Additionally, new 3-D inversion models have been performed by adding the new long-period data into the previous resistivity model of the island, performed prior to the 2021 volcanic eruption^13^. These new models provide novel and valuable information about the spatiotemporal evolution of the electrical resistivity, which will play a key role in understanding the geological significance and implications of these geoelectrical fluctuations.

The findings of this study highlight the strong potential of the MT method as a volcanic monitoring tool. Combining data from different geophysical techniques is critical to gain a comprehensive understanding of physical processes and contribute to hazard mitigation. Therefore, the consideration of new monitoring techniques that provide additional types of data would be highly beneficial.

## Results

### MT data: Electrical resistivity changes over time

The original time series analyzed here, which spanned over a period of more than fourteen months, were divided and processed into segments of ten days each. This segmentation resulted in a set of forty-five MT curves named LP1 to LP45 (Fig. 1B and supplementary Figs. SM1 and SM2). This allowed to obtain magnetotelluric impedance tensors that cover the 30–10,000 s period range.

The first results obtained reveal significant changes in the magnetotelluric impedance tensor within days, mainly for the Z_xy_ component (Fig. 1B,C and supplementary Fig. SM3). Overall, the data quality is good, except for the impedances estimated from the data acquired during the syn-eruptive stage (05.11.2021–13.12.2021), which show a noticeable decrease in quality (light grey in supplementary Fig. SM3) likely due to the noise induced by the volcanic activity itself. Additionally, MT curves from LP28 to LP39 are not presented here due to the impossibility to obtain accurate estimates during that period caused by damage to one of the non-polarizable electrodes.

**Figure 2:**
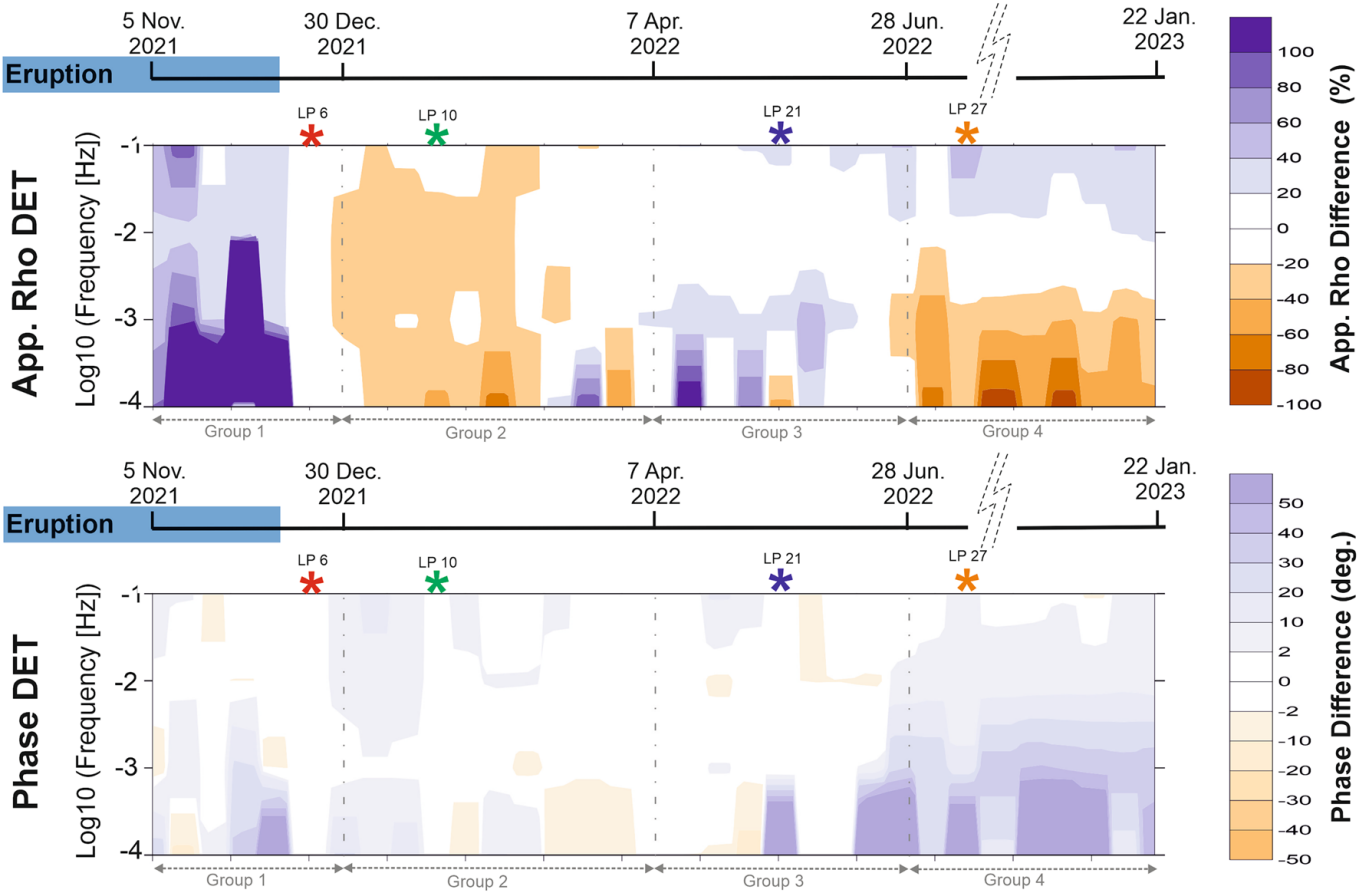
Pseudosections of the apparent resistivity and phase determinant obtained by subtracting the reference MT curve (LP6) from the observed dataset. The horizontal axis corresponds to time. Stars: Selected MT curves LP6, LP10, LP21 and LP27, used to perform the new inversions. Grey dashed lines: Four groups of resistivity curves into which the dataset can be divided.

Given the significant differences observed in the Z_xy_ component, and in order to understand the behavior of the full impedance tensor over time, the data were represented in the form of pseudosections. Figure SM4 (in the supplementary material) shows the pseudosections of each of the four components individually, while Fig. 2 shows the temporal evolution of the electrical resistivity by plotting the apparent resistivity and the phase determinant. The plot represents the difference between the observed dataset and the LP6 MT determinant, which was selected as a reference curve for being the first one of the post-eruptive stage (from 13.12.2021) with adequate data quality (Fig. 1C and supplementary Fig. SM3).

Following the volcanic eruption, the dataset can be divided into four major temporal groups of MT apparent resistivity curves (Fig. 2 and supplementary Fig. SM3). The first group corresponds to the immediate period after the volcanic eruption and is represented by the baseline MT curve LP6. From this point, a clear and gradual trend toward lower resistivity values is observed, causing the apparent resistivity curves to shift gradually to more conductive values over time (Figs. 1C and 2). The second group, represented by the MT curve LP10, shows a relative decrease in electrical resistivity of over 20% across the entire period range compared to the baseline curve (Fig. 2). Starting from 08.04.2022, the third group of MT curves represented by LP21 exhibits a relative increase in electrical resistivity of over 20% at long periods. Finally, the fourth group (represented by the MT curve LP27) demonstrates a distinct behavior. In this case, the apparent resistivity curves exhibit a sharper morphology compared to the previous groups, with an even larger relative increase in resistivity values from the baseline curve (Figs. 1C and 2). However, the determinant pseudosections indicate a decrease in resistivity at long periods, accompanied by phase differences exceeding 2 degrees across the entire period range (up to more than 30 degrees at long periods, Fig. 2). In the last period of time (fourth group of temporal MT curves), slight changes in the Zyx and Zyy components are also observed (Supplementary Fig. SM4), whereas the earlier electrical resistivity variations predominantly affected the components involving the N-S electric field (Zxy and Zxx).

These observations suggest that, for most of the analyzed time period, the temporal changes observed in the MT responses are likely associated with the behavior of the N-S electrical field (Ex). Based on this observation, the recorded magnetic field was compared with the synchronous data from the nearest geomagnetic observatory located in Güimar (Tenerife Island, 145 km away). The good agreement between the variations observed in both datasets indicates that the horizontal components of the magnetic field remain largely unaffected by the volcanic activity, supporting the idea that the electric field is the main cause of the observed changes (Supplementary Fig. SM5. Güimar data from Intermagnet). An exhaustive analysis of the time series recorded during and after the volcanic eruption is undoubtedly necessary to fully understand the origin, meaning and implications of the EM field behaviour. Nevertheless, the initial examination performed here points to variations on the N-S electric field as the primary cause of the resistivity changes observed in the post-eruptive stage.

### ***3-D resistivity models: ***Spatiotemporal*** evolution of the electrical resistivity***

The pre-eruptive geoelectrical structure of La Palma Island had been characterised by a previous 3-D MT model^13^, which can be used as starting model to assess the spatiotemporal evolution of the electrical resistivity in the post-eruptive stage.

The estimated impedances selected as representative for the four temporal MT groups mentioned earlier (LP6, LP10, LP21 and LP27; Figs. 1C, 2 and supplementary Fig. SM3) have been used to perform new 3-D inversions, since these correspond to the major temporal changes observed in the electrical resistivity.

**Figure 3:**
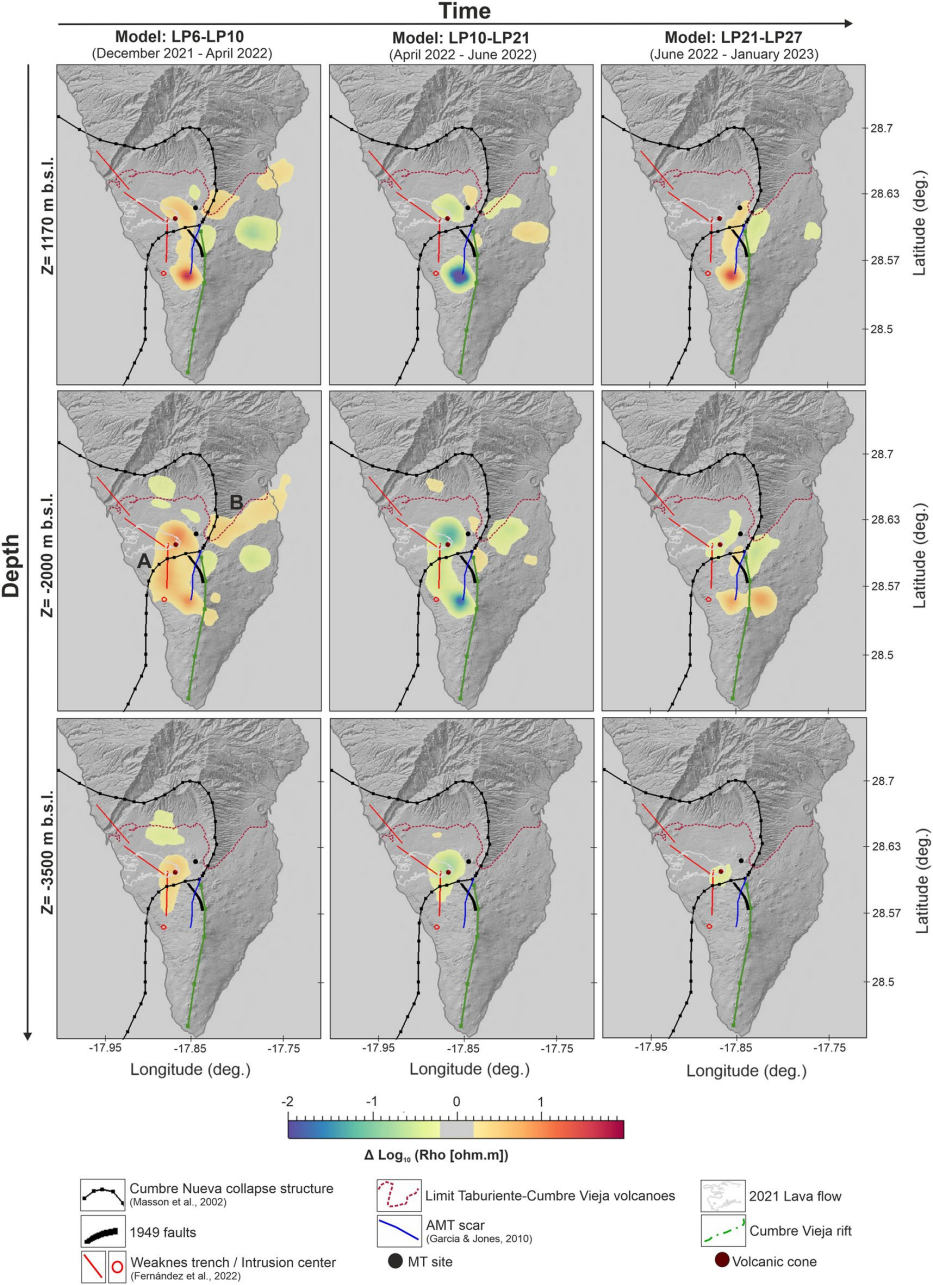
Map views at different depths showing the logarithmic difference between one 3-D resistivity model and the subsequent one. (**A**) and (**B**): major temporal alignments of resistivity anomalies. Matlab software R2021a (www. mathworks. com), Geoscience ANALYST (https://mirageoscience.com/mining-industry-software/geoscience-analyst/) and Microsoft PowerPoint 2019 were used to create this figure.

**Figure 4:**
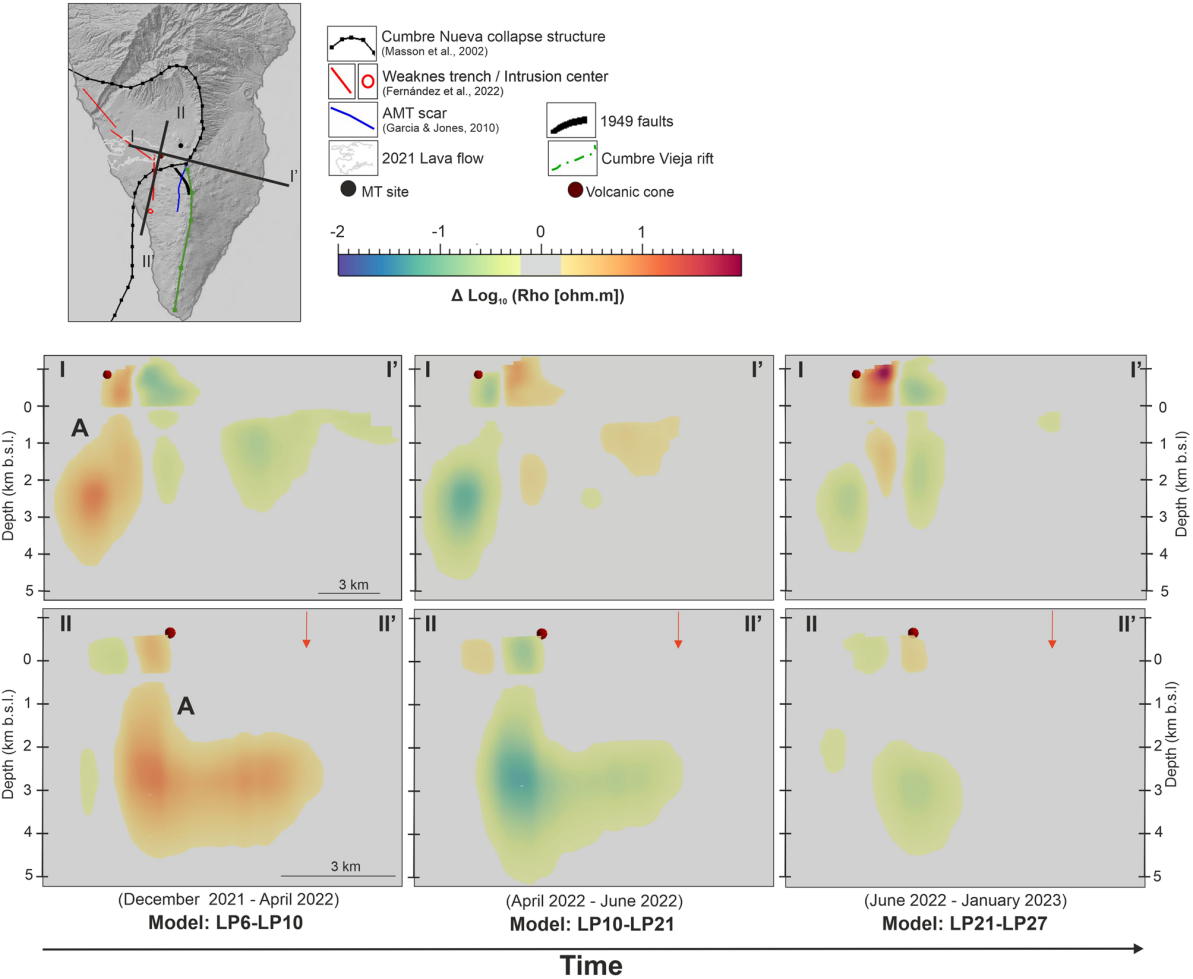
Vertical cross-section across the 2021 Cumbre Vieja volcanic cone (red sphere) showing the logarithmic difference between a 3-D resistivity model and the subsequent one. Red arrow: location of the intrusion center proposed by^14^. Matlab software R2021a (www. mathworks. com), Geoscience ANALYST (https://mirageoscience.com/mining-industry-software/geoscience-analyst/) and Microsoft PowerPoint 2019 were used to create this figure.

**Figure 5:**
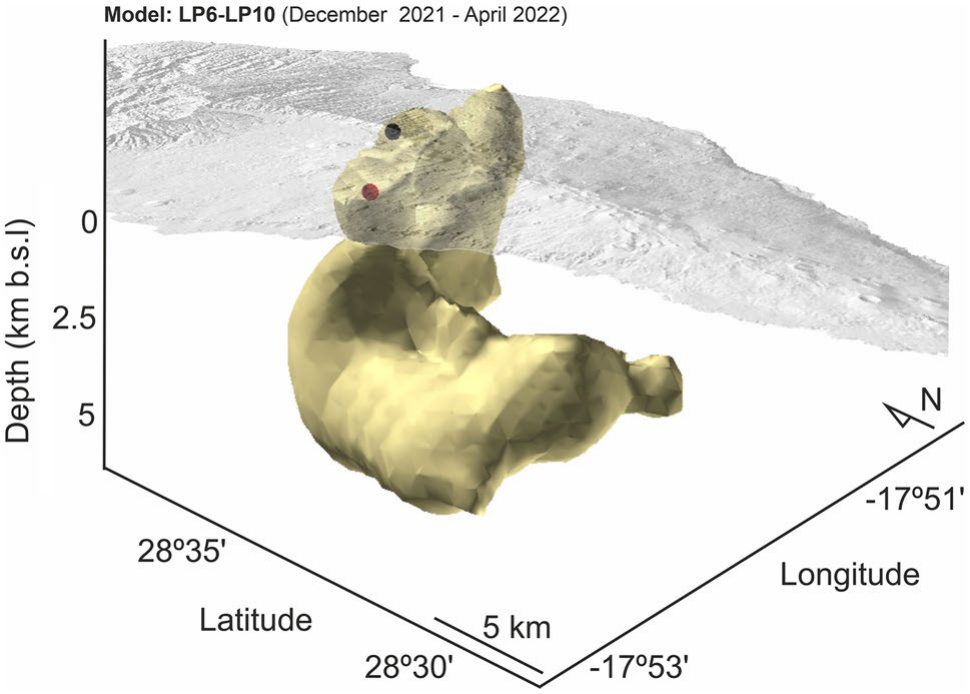
Three-dimensional view of the LP6-LP10 model. The volume corresponds to logarithmic resistivity differences between 0.2 and 2. Red sphere: 2021 Cumbre Vieja volcanic cone; Black sphere: Location of the long period MT site.

Consequently, four new 3-D resistivity models were obtained by individually inverting each of the selected MT curves. At first sight, the resulting resistivity models appear to exhibit no significant differences among them (Supplementary Fig. SM6). However, closer inspection reveals conspicuous differences. Figures 3, 4 and 5 show different views of the resultant 3-D resistivity volumes obtained by subtracting one model from the subsequent one (i.e. model LP6—model LP10; model LP10—model LP21; model LP21—model LP27).

The most significant changes in resistivity occur in the central-western part of the island at depths ranging from 1 to 4 km below sea level (b.s.l.). In the early post-eruptive stage (Model LP6-LP10; December 2021-April 2022), a clear decrease in electrical resistivity over time is observed, with the major changes occurring along two well developed alignments (Fig. 3, left panel).

The first alignment, labeled "A" in Figs. 3 and 4, lies beneath the 2021 Cumbre Vieja volcano and extends southward with an overall north–south trend. This anomaly is in good agreement with one of the alignments of minimum density associated with shallow magma storage as interpreted from the recent InSAR studies conducted on the island^14^ and it appears to be constrained to the east by a fault line inferred in a previous magnetotelluric study conducted on the island^15^. Notably, the largest resistivity variations are observed precisely at the southern end of this inferred fault. This indicates a slight eastward turn in the direction of the temporal resistivity anomaly with depth, in agreement with the shallow dike-related seismicity reported by^16^. The strong vertical contrasts observed in the resistivity changes near the volcanic cone (Fig. 4) are supported by the seismic tomography model performed by^17^, which shows significant differences in both P-wave and S-wave anomalies in these same areas with a similar vertical pattern at shallow depths. The vertical low-velocity anomaly identified in this study aligns with the location and trend of the temporal resistivity anomaly "A" and was interpreted by the authors as a zone of structural weakness that may have favoured the magma ascent.

The second alignment, labeled "B" in Fig. 3, is particularly prominent during the early post-eruptive stage at depths around 2 km b.s.l. It appears to be correlated in some way with the boundary between the oldest and youngest parts of the island (Taburiente and Cumbre Vieja volcanoes). A similar trend is observed in S-wave velocities^17^. Figure 5 provides a three-dimensional view of the areas where electrical resistivity decreases with time during this early post-eruptive stage.

From April to June 2022 (Model LP10-LP21), the same temporal resistivity anomalies are observed, but with the opposite sign. This means that the electrical resistivity increases over time in areas "A" and "B" (Figs. 3 and 4, central panels). Starting from June (Model LP21-LP27; Figs. 3 and 4, right panels), there is a significant reduction in the size of the temporal resistivity anomalies. Anomaly "B" is no longer visible, and the anomaly "A" at shallow depth is no longer found beneath the 2021 Cumbre Vieja volcano and the N-S structural weakness zones^14^. Instead, it appears to be correlated with the fault inferred by^15^ and the Cumbre Nueva landslide wall.

## Discussion

Electrical resistivity in the proximities of the volcanic cone varies by orders of magnitude within days. Such behaviour can only be triggered by changes on the subsoil of great significance, since at this scale of work MT responses are expected to remain largely unchanged with time. However, MT responses are very sensitive to the presence of fluids, which content, composition or distribution can change relatively quickly in the subsoil.

Therefore, we consider that these temporal changes in resistivity are more plausibly explained by changes in fluids content and/or their characteristics driven by the volcanic activity. This explanation is supported by the strong correlation between our findings and the seismic evidences of fractured zones that would offer low resistance to fluids migration.

**Figure 6:**
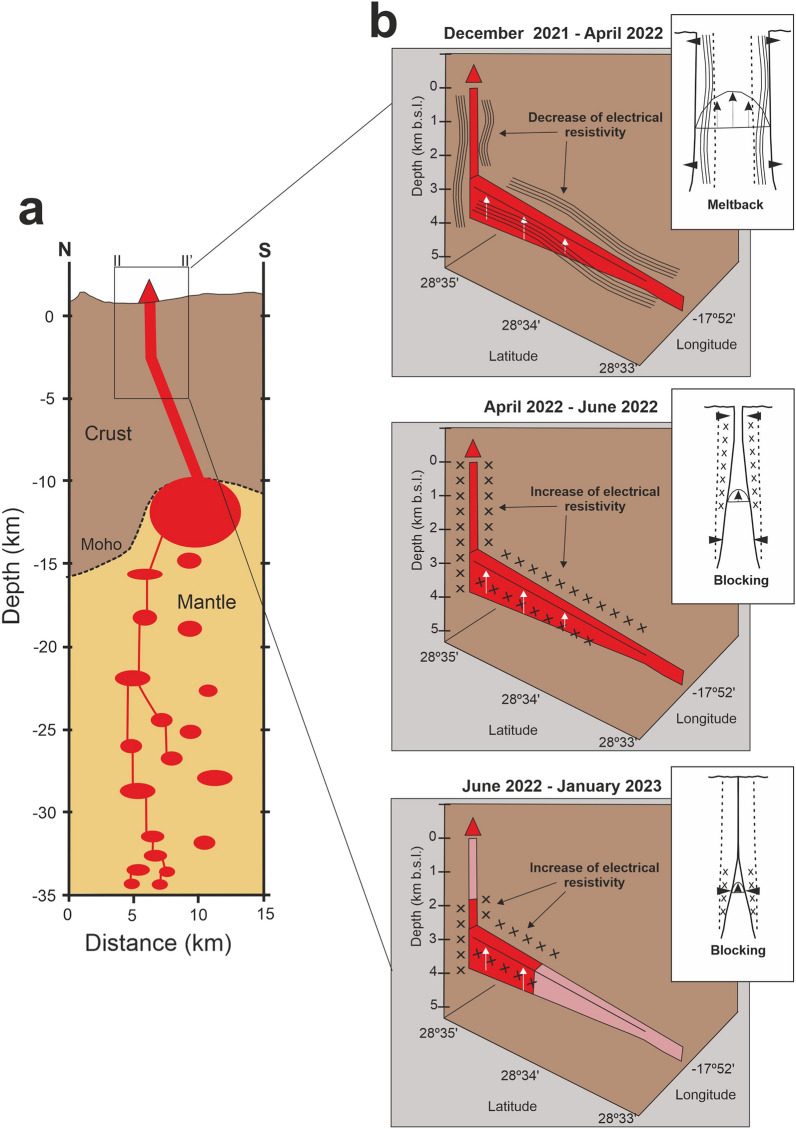
Interpretation of spatiotemporal changes in electrical resistivity during the post-eruptive stage. (**a**) Magmatic plumbing system interpreted from seismic data^16,17^. (**b**) Evolution of shallow magmatic ascent pathways (5 km depth) along vertical cross-section II-II’ as in Fig. 4. Meltback and blocking sketch after^21^. Pink color in bottom-right panel: area with no significant changes in electrical resistivity during that period of time.

Although no continuous MT data was recorded during the pre-eruptive stage and data quality during the syn-eruptive stage is inevitably impacted by the volcanic eruption itself, the post-eruptive dataset has proven to be a valuable source of information:

- **(1)** Temporal variations of more than 20% in apparent resistivity and 2 degrees in phase have been detected with a fourteen months length dataset. This is the largest long-period MT dataset recorded continuously during and after an active volcanic eruption published to date. These characteristics making it a unique and highly valuable resource for evaluating the potential of the MT method as a volcanic monitoring tool.
- One of the most interesting aspects of this dataset is the fact that not all impedance components change over time in the same way. Under the measurement directions (impedance tensor rotation equal to zero), only those components involving the E_x_ electric field are significantly changing with time (Fig. 1B,C ). This corresponds to the N-S direction, which in the southern part of the island has very important geological implications. The Cumbre Vieja rift (Fig. 1A), a well developed north-south trending rift zone, is controlling the geology of this area of the island (e.g.^18,19^), and it may be conditioning to a large extent the long-period MT responses.
- (2) The new MT inversion models provide novel key results on the spatial and temporal evolution of the subsoil electrical resistivity related to volcanic activity. The particular trace of the major temporal resistivity anomalies, in very good agreement with some previous interpreted structures^15^ and the most recent geophysical models^14,16,17^, indicates a clear structural control on the resistivities distribution linked to predefined fluids pathways.
- Temporal changes in electrical resistivity primarily occur along a well-defined north-south alignment situated on the western flank of Cumbre Vieja (Fig. 3). This area has recently been identified as a lineament of weakness due to its low density and the presence of velocity tomography anomalies, which facilitated magma ascent to the surface^14,17^. The distinctive vertical pattern observed in the temporal resistivity anomaly beneath the volcanic cone (Fig. 4) is consistent with the seismic activity associated with a shallow dike^16^, reinforcing the understanding of the vertical pathway for magma ascent in this region.
- (3) The spatiotemporal variations in resistivity provide valuable insights into the evolution of the dike intrusion during the post-eruptive stage, which has important implications for advancing our understanding of volcanic fluid pathways. Over a period of more than fourteen months, the area surrounding the shallow dike and the north–south alignment (structure labeled "A" in Figs. 3 and 4) have exhibited both, decrease and increase in electrical resistivity. Following the emplacement of a new dike feeding a fissure eruption, the process of magma cooling begins during which two different regimes can occur: blocking and meltback^20,21^. The first regime leads to the magma solidification until the dike becomes blocked, while during the second one, the surrounding country rock can be melted before the magma fully crystallizes^20,21^. The width of the dike is a particularly crucial parameter in determining the extent of each regime, such that large dikes can sustain significant meltback even without additional magma injections (static body of magma^20^). Considering an scenario with a static mass of magma having an initial temperature of ~ 1200ºC^22^ and a shallow dike with a width of around 7 m^23^ and more than 3 km length (this study), a little meltback months after the emplacement may be possible^20^. This interpretation is consistent with the spatiotemporal evolution of the geoelectrical properties of the magma ascent pathways, where the initial decrease in electrical resistivity could be related to the melting of the surrounding country rock (meltback, Fig. 6). The subsequent increase in resistivity would then be associated to the magma solidification along the walls of the dike (blocking, Fig. 6). Temperature of the country rock, dyke shape changes during magma cooling, further reinjection of magma, etc.… are important parameters controlling the extent of the blocking and meltback regimes^20^. The lack of information in this regard leaves several unanswered questions concerning the interpretation of the electrical resistivity changes attributed to the cooling process. However, the possibility of metlback seems to be a plausible interpretation for the decrease in resistivity during the initial 3.5 months following the volcanic eruption.
- To further advance the understanding of the post-eruptive evolution of the magma ascent pathway, MT data must be fully integrated with other geophysical and geochemical datasets.

The results here shown highlights the strong potential of the MT as a volcanic monitoring tool. In this particular case the monitoring activity is taken place in the post-eruptive stage but results obtained evidenced the capabilities of the MT to detect changes related to the magmatic system evolution, which suggests that the detection of similar changes during the pre-eruptive stage may also be possible.

Overall, the new 3-D resistivity models have allowed us to characterize a set of structures acting as preferred fluid pathways, with the most prominent being the one that facilitated the magmatic ascent during the 2021 eruption. Consequently, the other similar structures characterized here could potentially give rise to new eruptive centers in the future.

Any additional information which may assist in the volcanic monitoring task should be taken into account due to the important implications this type of activity has for the population and the volcanic hazard reduction. In this regard, 2021 La Palma long-period MT dataset seems a promising starting point for the critical assessment of the MT as a potential volcanic monitoring tool.

## Methods

### MT data

The dataset presented here corresponds to a new single long-period MT site located in the central part of the island, approximately 2.4 km away from the volcanic cone (Fig. 1A). The data was recorded using a LEMI-417 system (LVIV Centre of Institute of Space Research), which includes a three-component fluxgate magnetometer for registering the magnetic field. The electric field was measured using Pb/PbCl non-polarizable electrodes, and regular maintenance was performed approximately every three months. A long and continuous time series (4 Hz sampling) has been obtained since November 5, 2021, providing us with the record of over fourteen months that is analyzed in this study.

To work with such long measures and in order to check for changes on the electrical resistivity with time, the original time series was divided into ten-day packages. Working with ten days time series is reliable for both computational resources and processing accuracy, since these files are easy to handle but also have a proper number of sampling points to ensure the correct statistical estimation of the MT transfer functions. Consequently, each ten-day package was processed individually using the PRC_MTMV software^24,25^ following the same scheme for all packages. During the data processing, several tests were performed by overlapping the selected days, allowing the ten-day packages to have up to eight days in common. This approach enables the resultant apparent resistivity curves to capture changes occurring on a timescale shorter than ten days. Although more detailed images were obtained, the results consistently demonstrate the same major resistivity changes over time (Supplementary Fig. SM7).

For data representation, the apparent resistivity and phase curves themselves show the significant changes in electrical resistivity that occur within days (Figs. 1 and Supplementary Fig. SM3). SM3itionally, pseudosection plots of all four impedance tensor components, as well as the apparent resistivity and phase determinant, have been generated to analyse the results (Supplementary Fig. SM4 and Fig. 2). To create the pseudosection plots of the apparent resistivity and phase determinant, a selected reference temporal MT curve (LP6) was subtracted from the observed dataset.

### 3-D inversion models

The new 3-D inversion models were performed using the ModEM code^26,27^, the same one used to carry out the 3-D model previously performed by^13^. All models had the same grid discretization (120 × 88x83 cells), with an average resolution of 500 × 500 m horizontally in the core of the grid. In the vertical direction, the grid starts with an alternation of fine layers (from 100 to 10 m) to discretize the steep topography of the island. Bathymetry and ocean are also included in the models. Edges were padded with logarithmically increasing cells, so the final extension of the grid is 631 × 621x458 km.

None of the broadband MT sites recorded before the 2021 volcanic eruption have been included in this new set of models, in order to avoid possible conflicts between the new and the previous MT data due to the changes of the geoelectrical structure with time inferred during the data analysis.

All the inversions were undertaken by including only one temporal MT site at once (LP6, LP10, LP21 or LP27), with the full impedance tensor and tipper for 17 periods in the range from 30 to 10^4^ s. Starting model for all four new inversions was the previous 3-D resistivity model of the island^13^. After several tests and in order to assist the inversion process to get a more accurate result, both data and model grid were rotated according to the preferred geoelectrical strike direction at depth: N15ºE (e.g.^28^; Supplementary Fig. SM8).

During the initial stage of the inversion, most cells of the model were kept fixed at shallow depths, allowing modifications only in the southern area of the island, which was the region where the volcanic eruption-related earthquakes occurred^16^. This approach aimed to concentrate the inversion on the specific area of interest. In a subsequent stage of the inversion, the fixed area constraint was removed. Error floors were also modified several times during the inversion process. Initially we run the inversions with large error floor and then during the last stage the inversions were restarted using 10% error floor for all four components of the impedance tensor and tippers. The resulting root mean square (RMS) values for models LP6, LP10, LP21, and LP27 were 1.35, 1.30, 1.24, and 1.14 respectively, with an overall good data fit achieved. Supplementary Fig. SM9 shows the comparison of the observed and computed MT responses displayed as apparent resistivity phase and tippers curves.

The differences between the models shown in Figs. 3, 4, and 5 represent the logarithmic difference in resistivity between one model and the subsequent one (e.g., Model LP6-LP10 = Log_10_(LP6)—Log_10_(LP10)).

## Data availabiliy

The data supporting the findings of this study are publicly available online at: https://doi.org/10.34810/data749.

## Supplementary Information

Supplementary Information.
