# Supplementary material for: Volcanic monitoring of the 2021 La Palma eruption using long-period magnetotelluric data
Source: Sci Rep. 2023 Sep 23;13:15929. doi: 10.1038/s41598-023-43326-0 (PMC10517953; doi:10.1038/s41598-023-43326-0)
Supplement: Supplementary file 1 — Supplementary Information. [file 41598_2023_43326_MOESM1_ESM.pdf]

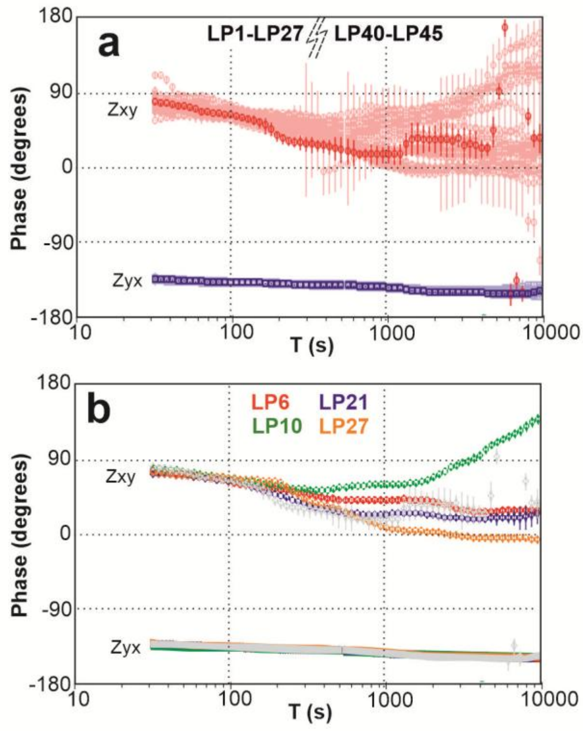

Figure SM1: A) Off-diagonal phase curves for the temporal MT impedances obtained by processing the original time series every ten days. Bright line: LP1 curve. B) Four selected apparent resistivity curves representative of the major temporal resistivity changes. Grey line: LP1 curve.

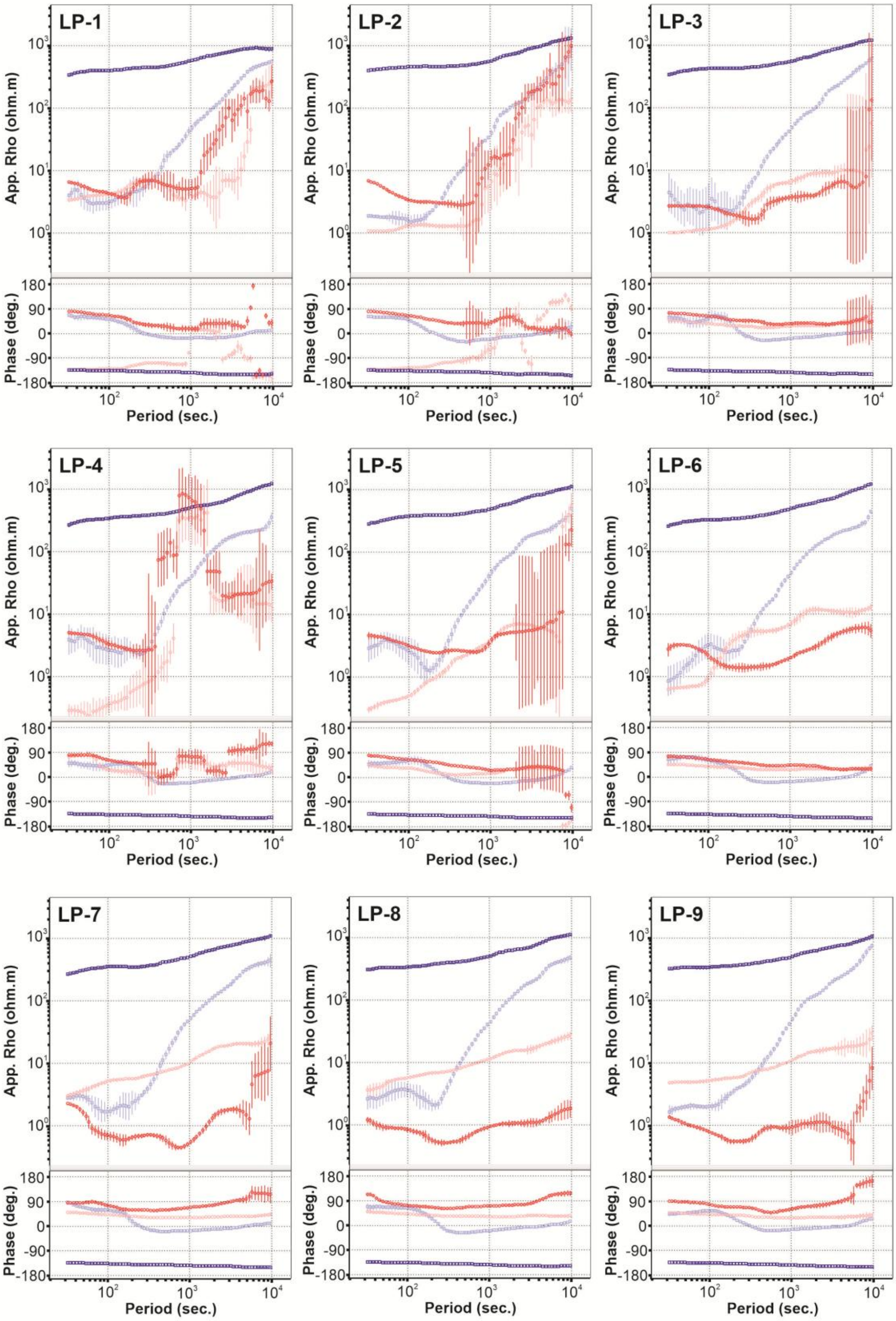

Figure SM2: Apparent resistivity and phase curves for the four components of the MT impedances obtained by processing the original time series every ten days. Rotation angle = 0 degrees.

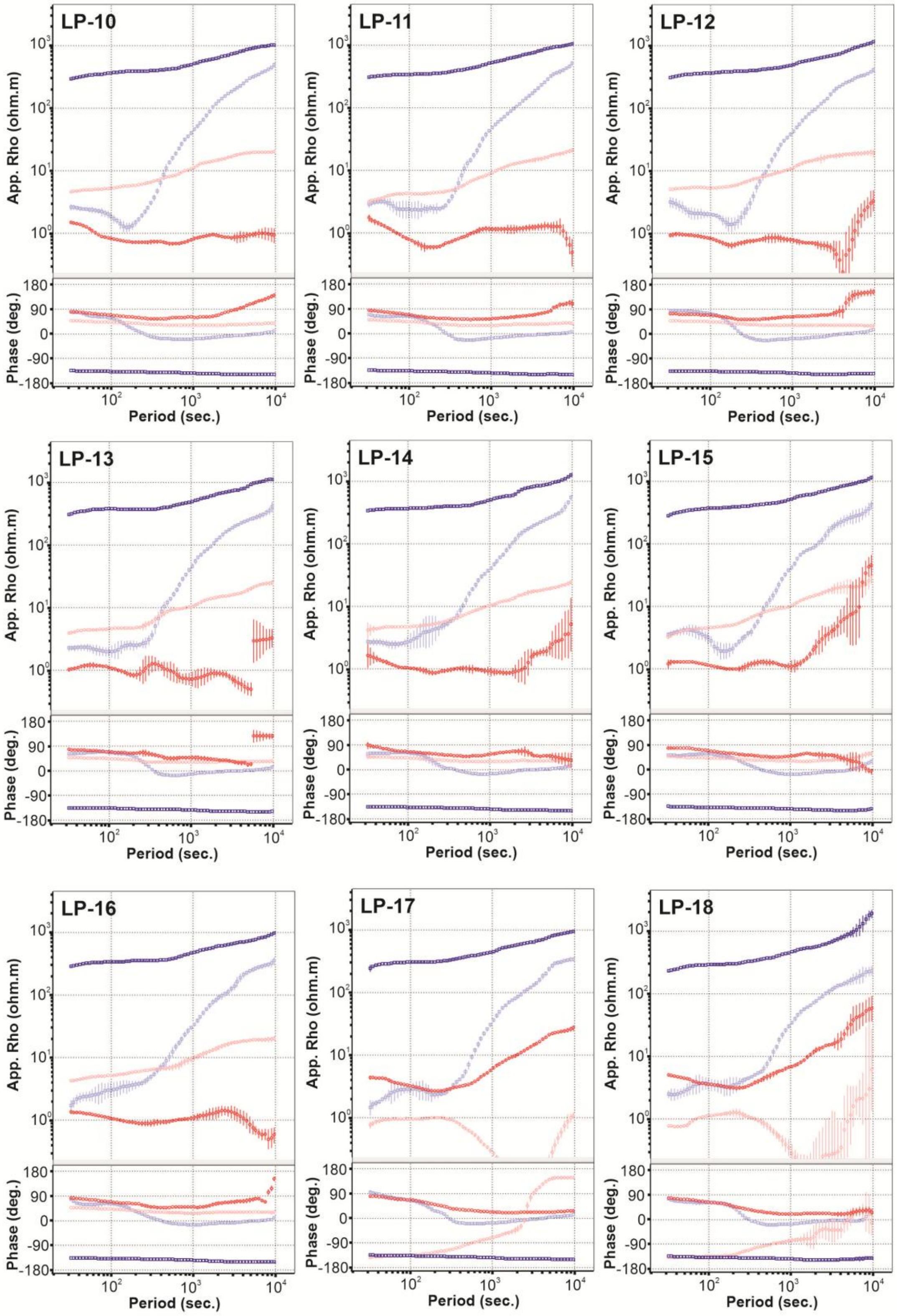

Figure SM2 (cont.)

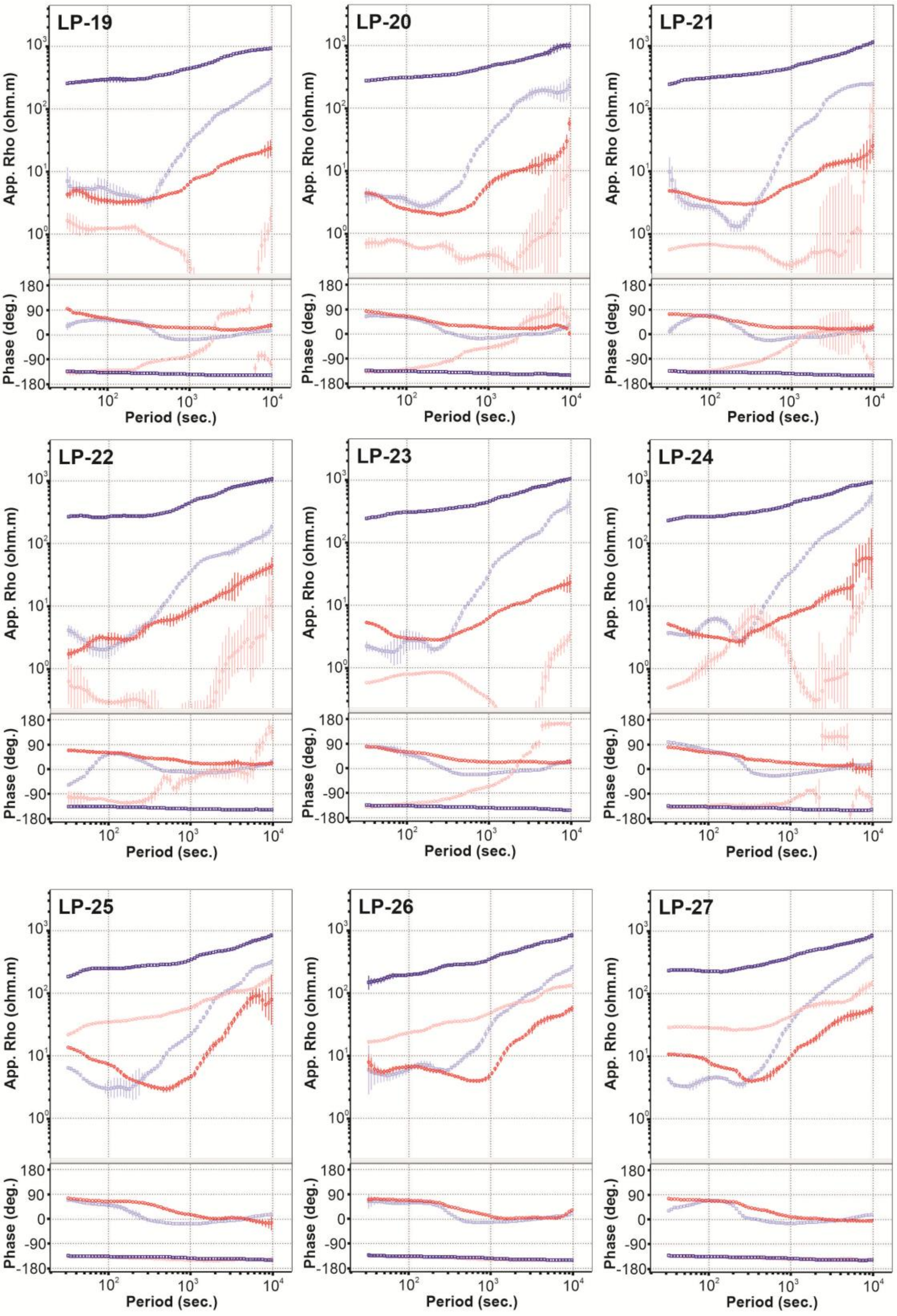

Figure SM2 (cont.)

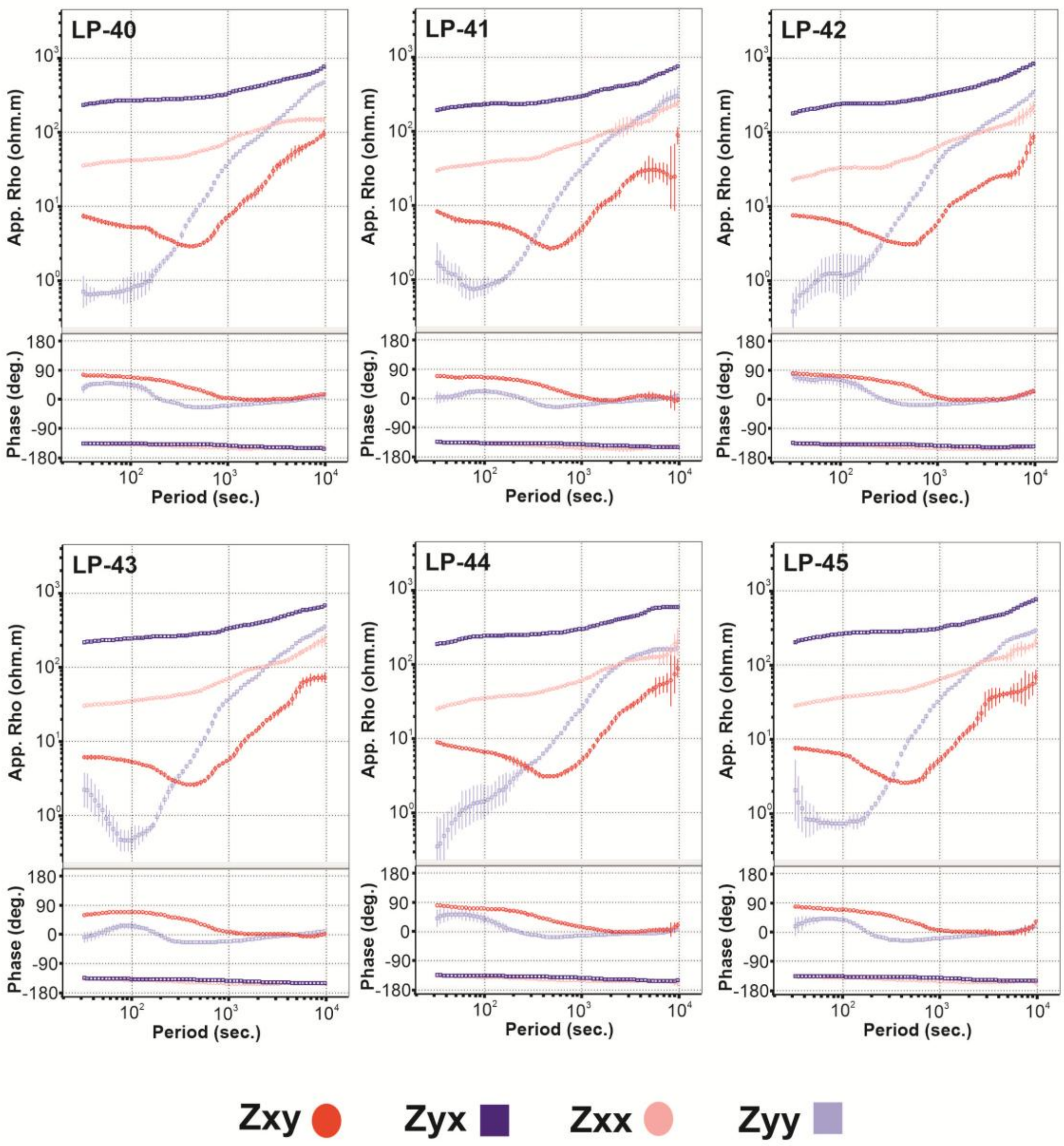

Figure SM2 (cont.)

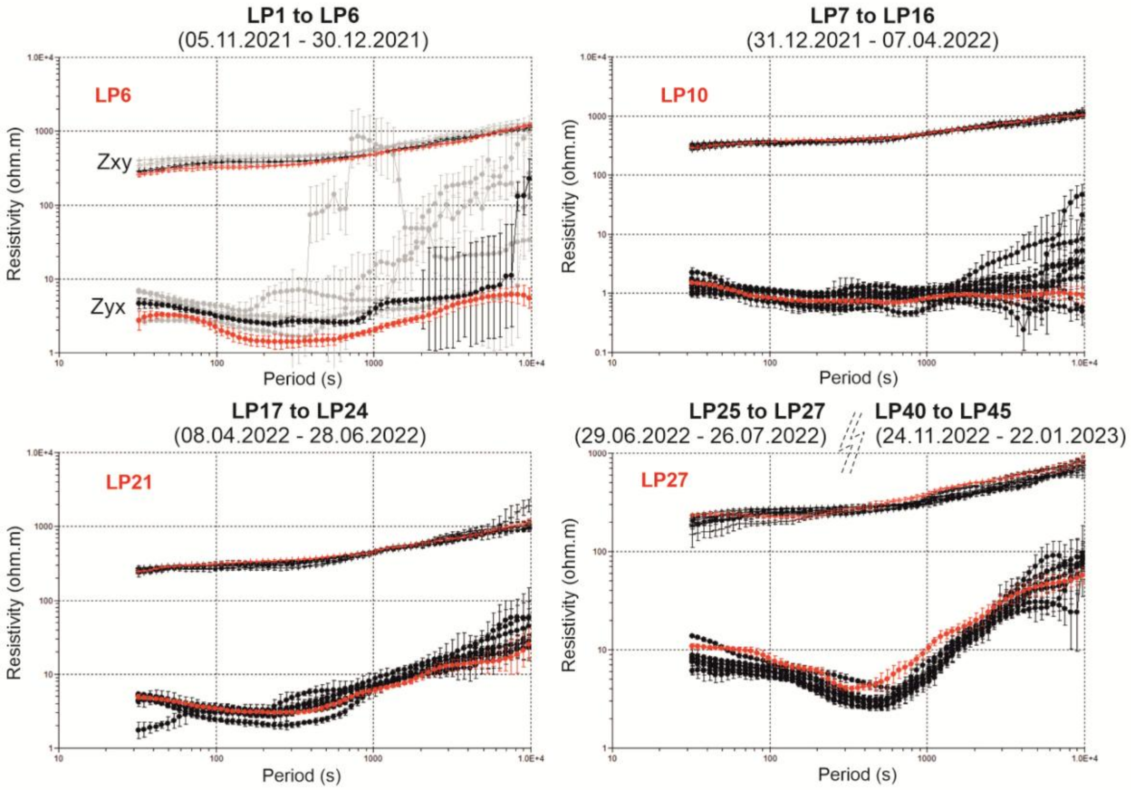

Figure SM3: Off-diagonal apparent resistivity curves grouped according to morphology. In red: curves selected as representative of each group and used to perform the new 3-D inversion models. In grey: curves LP1 to LP4, corresponding to the syn-eruptive stage.

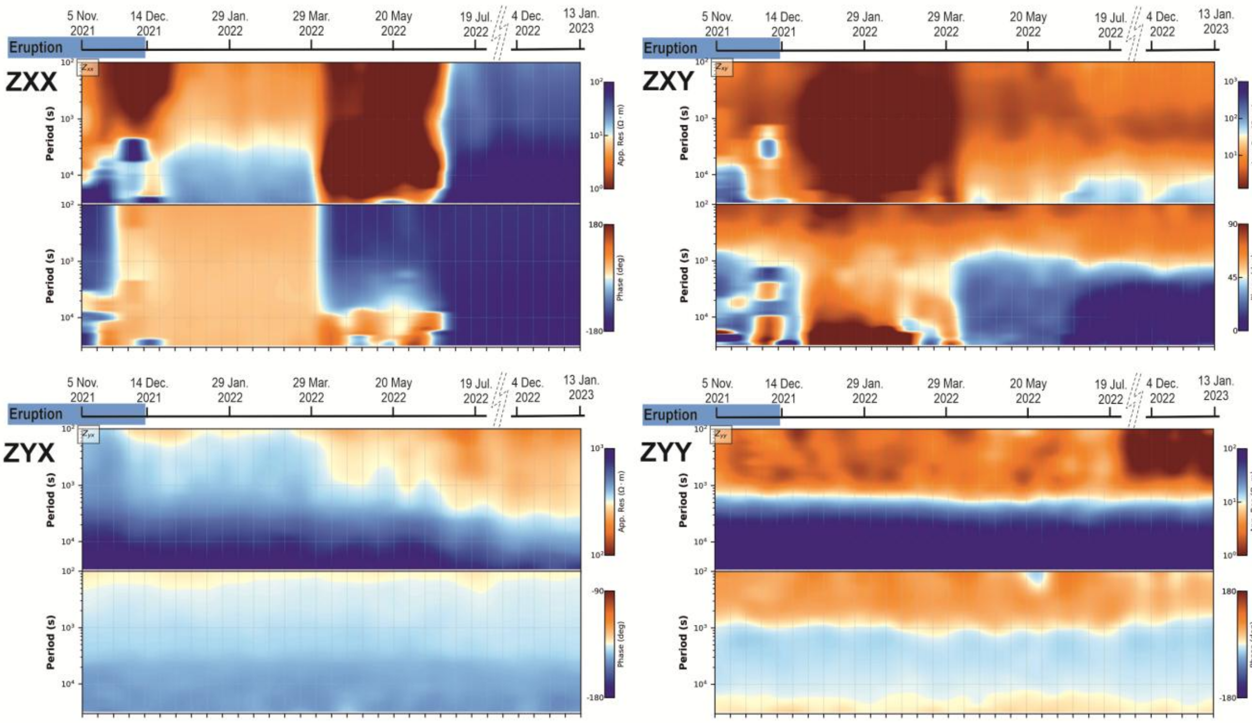

Figure SM4: Pseudosections of the apparent resistivity and phase for all four impedance tensor components. The horizontal axis represents time.

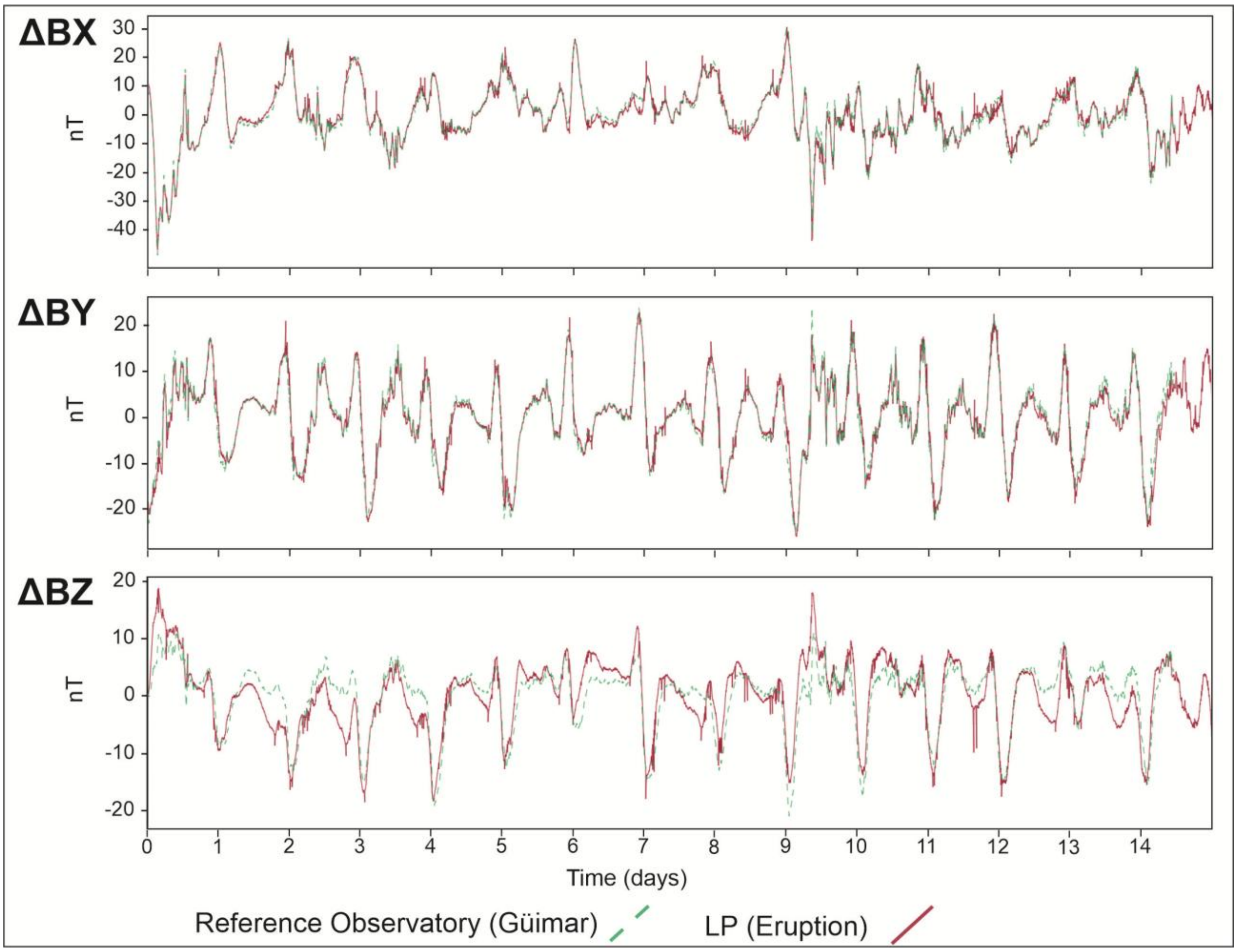

Figure SM5: Comparison of magnetic field variations recorded at the long-period MT site (red) and data from the Güimar observatory (green dashed line). Data recorded during the syn-eruptive stage (06.11.2021 to 20.11.2021).

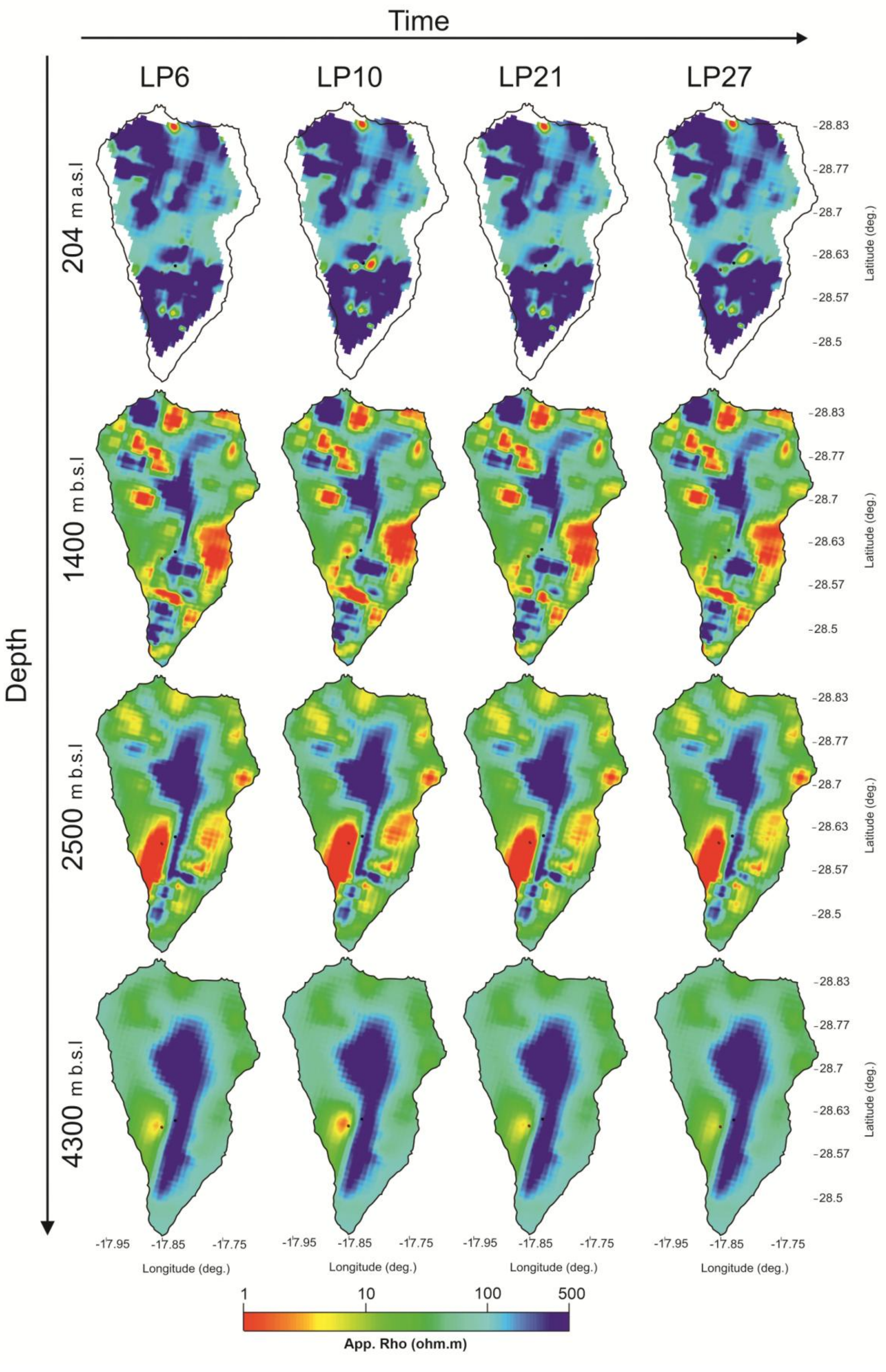

Figure SM6: Map views at different depths of the new 3-D models performed: LP6, LP10, LP21, and LP27. 3D-Grid Academic version 2.1.19 provided by Naser Meqbel

(Consulting-GEO: http://www.web3dmt.com/) and Microsoft PowerPoint 2019 were used to create this figure.

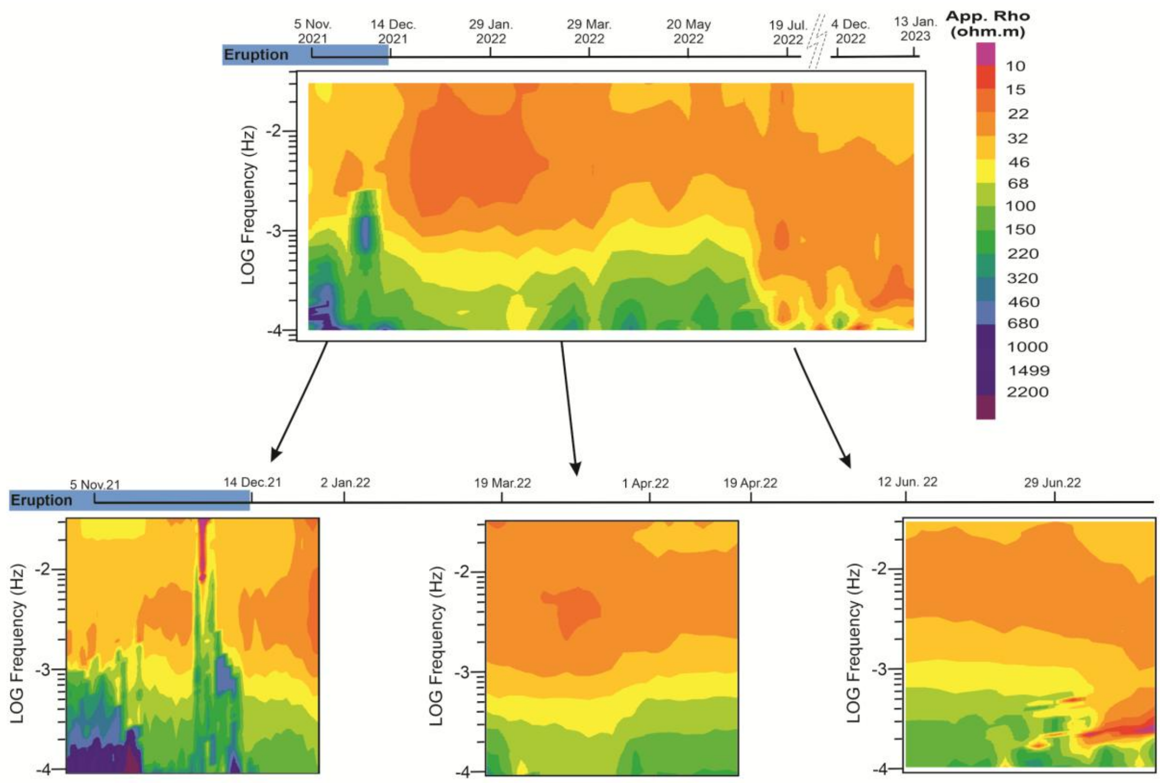

Figure SM7: Upper panel: Pseudosection of the apparent resistivity determinant for data processed every ten days without overlapping days. Lower panel: Pseudosections of the apparent resistivity determinant for specific time periods for data processed every ten days with eight days overlapping.

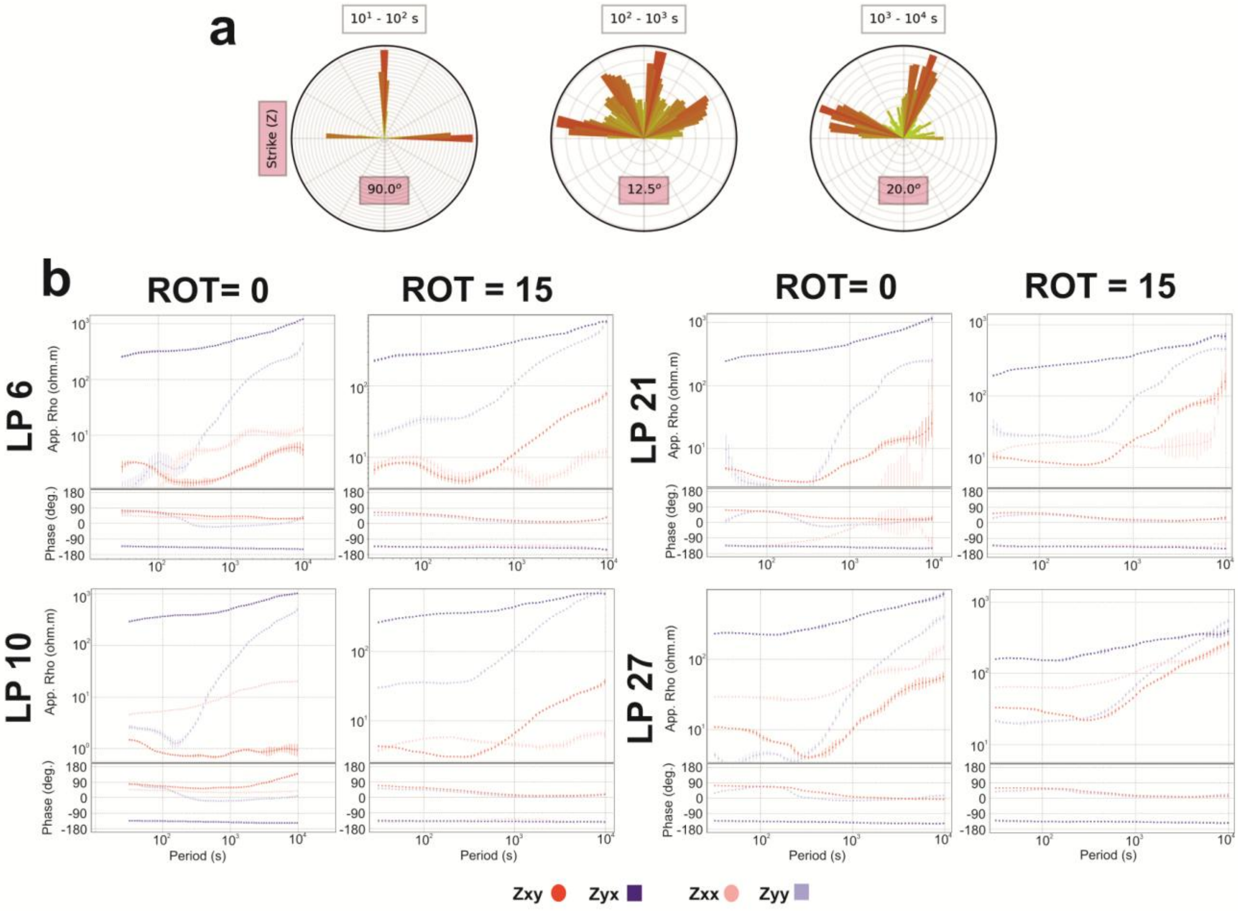

Figure SM8: A) Preferred strike direction obtained for three period bands (Computed using the MTpy package, Krieger and Peacock 2014). B) Apparent resistivity and phase curves of the four selected curves shown in Figure 1C for two different rotation angles: 0 and 15 degrees. Bright colors: off-diagonal components; Light colors: diagonal components.

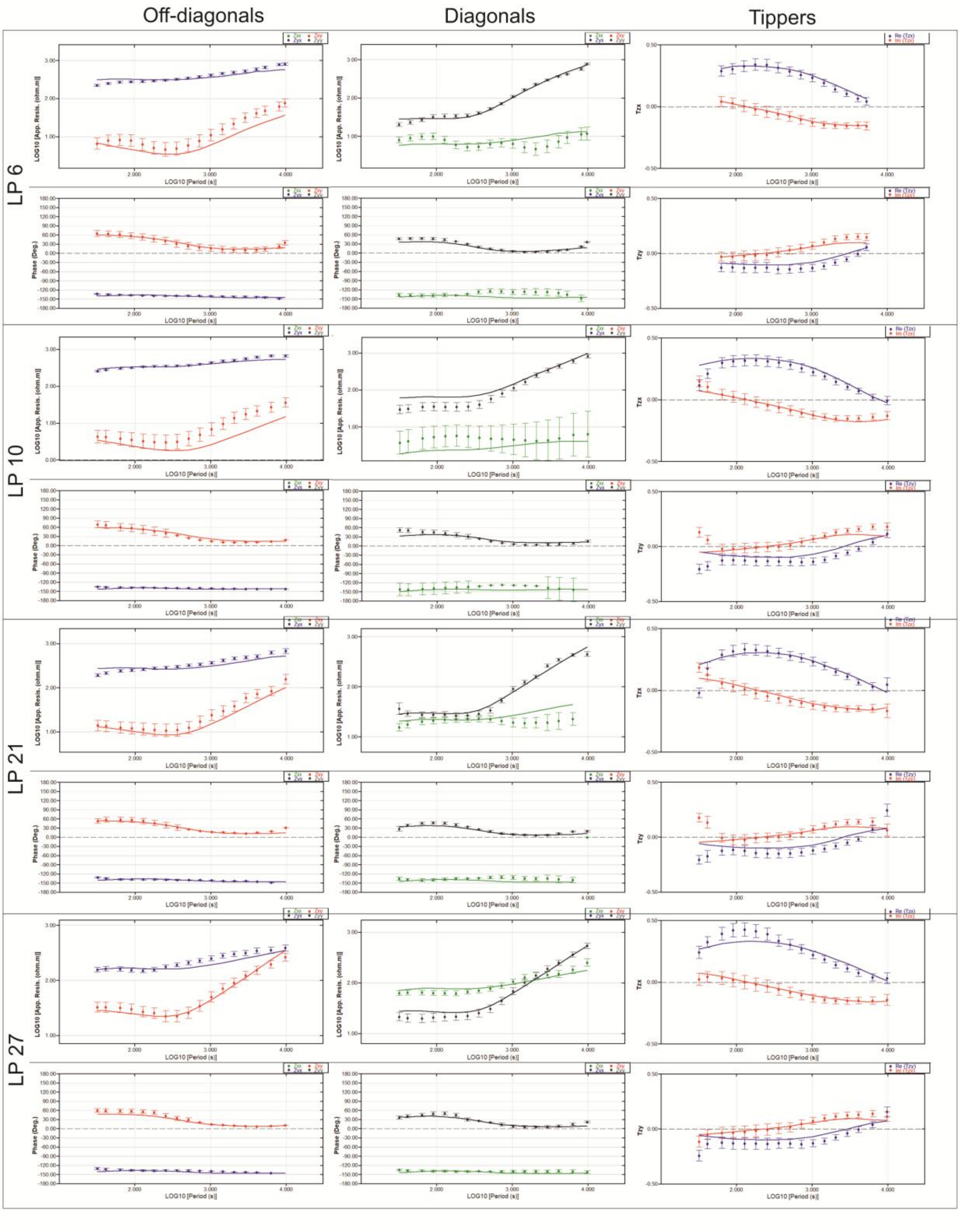
Off-diagonals
Diagonals
Tippers
LP 6
LP 10
LP 21
LP 27
LOG10 [App. Resis. (ohm.m)]
Phase (Deg.)
LOG10 [Period (s)]
Tzx
Tzy
Zxx
Zxy
Zyx
Zyy
Re (Tzx)
Im (Tzx)
Re (Tzy)
Im (Tzy)

Figure SM9: Comparison of the observed (dots) and computed (lines) MT responses for the four new inversions performed: LP6, LP10, LP21, and LP27.
